# Human and Porcine Hepatitis E Viruses, Southeastern Bolivia

**DOI:** 10.3201/eid1802.111453

**Published:** 2012-02

**Authors:** Michael A. Purdy, Maria Chiara Dell’Amico, José Luis Gonzales, Higinio Segundo, Francesco Tolari, Maurizio Mazzei, Alessandro Bartoloni, Yury E. Khudyakov

**Affiliations:** Centers for Disease Control and Prevention, Atlanta, Georgia USA (M. A. Purdy, Y. E. Khudyakov);; Università di Pisa, Pisa, Italy (M. C. Dell’Amico, F. Tolari, M. Mazzei);; Laboratorio de Investigación y Diagnóstico Veterinario, Santa Cruz, Bolivia (J. L. Gonzales);; Distrito de Salud Cordillera, Santa Cruz, Bolivia (H. Segundo);; Università degli Studi di Firenze, Firenze, Italy (A. Bartoloni)

**Keywords:** hepatitis E virus, HEV, viruses, Bolivia, human, swine, human-to-human transmission, genotype 3, most recent common ancestor, zoonoses

**To the Editor:** Hepatitis E virus (HEV) genotypes 3 and 4 are considered to be primarily zoonotic (*1*). However, recent data indicate that both genotypes can be transmitted among humans through other routes (*2**,**3*). Observations of genetic distinctiveness between swine and human HEV strains circulating within the same region argue against exclusivity of zoonotic transmission (*4*). A recent report presented a remarkable example of such distinction between genotype 3 isolates in rural communities in southeastern Bolivia (*5*).

We examined HEV sequences obtained in that study to show the independent genetic origin of swine and human variants. Findings suggest disjunction between human and swine HEV strains in this epidemiologic setting, despite the potential for extensive cross-species exposure.

Using reference sequences from Lu et al. (*6*), we conducted subtype analysis of HEV open reading frame 2 sequences at nucleotide positions 826–1173 (GenBank accession no. AF060668) from isolates from 2 rural communities in southeastern Bolivia (*5*). Analysis showed that swine sequences belonged to subtype 3i and that the human sequences belonged to 3e.

We collected all available GenBank genotype 3 sequences covering this genomic region for which the dates of collection were documented. Sequences were used to estimate the time from the most recent common ancestor (tMRCA) by using BEAST version 1.6.1 (*7*). Estimated tMRCA for GenBank sequences was longer than for sequences from Bolivia alone (Table) or for all genotype 3 sequences together (Table).

**Table:** Model estimates of time to most common recent ancestor for HEV ORF2 nucleotide sequences, southeastern Bolivia*

Model	Start position	Stop position	Bolivia sequence	GenBank sequence	Mean tMRCA, y	95% HPD, y		Mean ± SD rate of substitutions/site/y
						Lower	Upper	
A	826	1173	X	NU	55.31	16.26	108.05	3.27 × 10^–3^ ± 9.44 × 10^–6^
B	826	1173	NU	3i and 3e	148.63	3.01	291.46	1.88 × 10^–2^ ± 6.41 × 10^–4^
C	826	1173	X	X	144.45	38.66	298.20	3.43 × 10^–3^ ± 4.74 × 10^–5^
D	826	1173	NU	X	328.05	45.59	681.90	2.12 × 10^–3^ ± 7.33 × 10^–5^
E	1	1980	NU	X	296.06	163.40	467.97	9.27 × 10^–4^ ± 1.25 × 10^–5^
F	826	1173	3 seqs	X	275.45	40.06	635.32	2.40 × 10^–3^ ± 5.06 × 10^–5^

*HEV, hepatitis E virus; ORF, open reading frame; tMRCA, time to the most recent common ancestor; HPD, highest posterior density boundary; X, sequences was used; NU, not used; 3i and 3e, subtype 3i and 3e sequences were used; 3 seqs, sequences (CV2, CB9, and HB2BA053) from Bolivia were used.

To reduce the effect of close relatedness among human or swine HEV sequences from Bolivia on the tMRCA estimate, we used only 1 representative sequence per species from each community in the final analysis. This analysis identified an estimated tMRCA similar to that seen for GenBank sequences alone (Table, model F vs. model D). This estimate indicates that human and swine HEV isolates from southeastern Bolivia last shared a common ancestor ≈275 years ago (Table, model F). Thus, swine HEV strains from both rural communities belonged to subtype 3i, and the human HEV strains identified from the community of Bartolo, Bolivia, belonged to subtype 3e and shared an ancestor with swine strains almost 3 centuries ago.

This finding is surprising because the community of Bartolo has several potential risk factors for zoonotic transmission of HEV. There are ≈200 humans and ≈70 swine in Bartolo (*8*). Residents are mainly native Quechua and Guarani with some of mixed Spanish ancestry who subsist at a low socioeconomic level. Their main livelihood activities are agriculture and breeding of animals. Free-range pig farms are family owned. Because of its impoverished state, the community has no running water, and few houses have toilets. No facilities are suitable for safely slaughtering animals (*5**,**9*). These conditions appear to create a setting in which zoonotic transmission of HEV should be common, and infection should be caused by a strain shared between swine and humans. However, the data suggest host-specific infection with distinct HEV subtypes.

Although specimens were collected from 172 humans (≈86%) and 67 swine (≈96%) in Bartolo (*8*), zoonotically transmitted isolates may have been missed because of the sample-pooling technique used (*5*). Nevertheless, detection of distinct HEV strains in human and swine populations indicates possible nonzoonotic, human-to-human transmission in this community. Detection of antibodies against HEV among 7% of residents and HEV genomes in persons without serologic markers of HEV infection indicate a higher HEV prevalence in Bartolo (*5*). Subclinical infection detected by PCR among Bartolo residents (*5*), rapid decrease of HEV antibody, and uncertain sensitivity of commercial serologic assays (*10*) suggest that the reported extent of HEV infection is most likely an underestimate.

High prevalence may generate conditions in this community that effectively prevent cross-species transmission because of frequent exposure to HEV early in life when contacts between humans and animals are limited, thus promoting host-specific transmission. This supposition is supported by the higher seropositivity seen among children 1–5 years of age and adults 41–50 years of age in Bartolo (*5*). Implications of these observations for understanding HEV evolution and epidemiology of HEV infections warrant further research on genetic heterogeneity of HEV strains in this region and other epidemiologic settings.
